# Roles of RbcX in Carboxysome Biosynthesis in the Cyanobacterium *Synechococcus elongatus* PCC7942^1^

**DOI:** 10.1104/pp.18.01217

**Published:** 2018-11-02

**Authors:** Fang Huang, Olga Vasieva, Yaqi Sun, Matthew Faulkner, Gregory F. Dykes, Ziyu Zhao, Lu-Ning Liu

**Affiliations:** Institute of Integrative Biology, University of Liverpool, Liverpool L69 7ZB, United Kingdom

## Abstract

Rubisco is the essential enzyme mediating the fixation of atmospheric CO_2_ during photosynthesis. In cyanobacteria, Rubisco enzymes are densely packed and encapsulated in a specialized organelle known as the carboxysome. Well-defined Rubisco assembly and carboxysome formation are pivotal for efficient CO_2_ fixation. Numerous chaperone proteins, including RbcX, are essential for proper protein folding and Rubisco assembly. In this study, we investigated the in vivo function of RbcX in the cyanobacterium *Synechococcus elongatus* PCC 7942 (Syn7942) using molecular, biochemical, and live-cell fluorescence imaging approaches. Our results show that genetic deletion of the *rbcX* gene affects Rubisco abundance, as well as carboxysome formation and spatial distribution. Moreover, RbcX appears as one component of the carboxysome and shows a dynamic interaction with Rubisco enzymes. These in vivo observations provide insight into the role of RbcX from Syn7942 in mediating carboxysome assembly. Understanding the molecular mechanism underlying Rubisco assembly and carboxysome biogenesis will provide essential information required for engineering functional CO_2_-fixing complexes in heterogeneous organisms, especially plants, with the aim of boosting photosynthesis and agricultural productivity.

## Abstract (precis)

RbcX, the chaperone for Rubisco biogenesis, is involved in carboxysome assembly in Synechococcus elongatus 7942.

Rubisco catalyses the conversion of atmospheric CO_2_ into organic carbon biomass in photosynthesis and thus has profound implications for life on Earth. Among the distinct forms of Rubisco found in nature, form I Rubisco, comprising form IA and form IB types, is the most abundant in plants, algae, cyanobacteria, and proteobacteria (Tabita et al., 2008; Hauser et al., 2015b). It is an ∼550-kD hexadecamer complex containing eight Rubisco large subunits (RbcL, ∼53 kD) and eight Rubisco small subunits (RbcS, ∼15 kD), designated as RbcL_8_S_8_ (Andersson and Backlund, 2008; Bracher et al., 2017). The RbcL subunits are arranged as a tetramer of antiparallel RbcL dimers, and four RbcS subunits each cap the top and bottom. The assembly of the cyanobacterial form I Rubisco requires a number of auxiliary proteins. Folding of cyanobacterial RbcL is mediated by the chaperonin GroEL and its cofactor GroES (the homologs in plants are Cpn60 and Cpn20) and subsequently leads to the formation of a RbcL dimer (Hayer-Hartl et al., 2016). The stabilization of the RbcL dimer and further assembly of RbcL_8_ require specific assembly chaperones, including a homodimer of RbcX and a dimer of Rubisco accumulation factor1 (Raf1) (Saschenbrecker et al., 2007). In addition, Rubisco accumulation factor2 (Raf2) and the chloroplast-specific protein bundle-sheath defective2 (BSD2) have been characterized as important assembly chaperones at a late stage of Rubisco biogenesis in plants (Feiz et al., 2012; Wheatley et al., 2014; Hauser et al., 2015a; Aigner et al., 2017).

In most cyanobacteria, RbcX is the product of the *rbcX* gene that is commonly located in the same operon between the *rbcL* and *rbcS* genes, indicating its structural or functional relationship with Rubisco (Liu et al., 2010; Bracher et al., 2017; Hayer-Hartl, 2017). In the marine cyanobacterium *Synechococcus* sp. PCC7002 (Syn7002), partial inactivation of *rbcX* resulted in a significant reduction in Rubisco solubility and activity (Onizuka et al., 2004). RbcX from *Anabaena* sp. Strain carbonic anhydrase (CA) was found to enhance the expression and activity of recombinant Rubisco in *Escherichia coli* (Li and Tabita, 1997). Structural analysis of RbcX from Syn7002 revealed its function in promoting the formation of the RbcL_8_ core following the RbcL folding, by interacting with RbcL binding domains (Saschenbrecker et al., 2007). Previous studies on the structure of the RbcL_8_-(RbcX_2_)_8_ assembly intermediate further demonstrated that RbcX functions in stabilizing the RbcL dimer and facilitating RbcL_8_ assembly (Liu et al., 2010). By contrast, the *rbcX* genes in the freshwater unicellular cyanobacteria *Synechococcus elongatus* sp. PCC7942 (Syn7942) and *Synechococcus elongatus* PCC6301 (Syn6301) are >100 kb away from the Rubisco *rbcLS* operon, indicative of the functional specificity of RbcX in these species. Inactivation of *rbcX* in Syn7942 by interrupting its coding sequence had no significant effect on cell growth (Emlyn-Jones et al., 2006b). Likewise, RbcX was found not necessary for the assembly of engineered cognate Syn7942 Rubisco in tobacco (*Nicotiana tabacum*) chloroplasts (Occhialini et al., 2016). The exact physiological significance of RbcX in Syn7942 cells is still enigmatic.

Despite its essential role in photosynthetic carbon fixation, Rubisco is an inefficient enzyme, ascribed to its slow catalytic rate and restricted capability in discriminating between CO_2_ and O_2_ as the substrate. To suppress the oxygenase reaction and enhance the carboxylation of Rubisco enzymes, cyanobacteria have evolved the specialized bacterial microcompartments, the carboxysomes, as the central part of CO_2_-concentrating mechanisms (CCMs; Rae et al., 2012; Kerfeld and Melnicki, 2016). There are α-type (containing form IA Rubisco) and β-type (containing form IB Rubisco) carboxysomes. In β-carboxysomes, form IB Rubisco and CA are densely packed into an ordered matrix with internal linker proteins to form the enzyme core, which is encapsulated by a proteinaceous shell (Long et al., 2007; Cameron et al., 2013; Faulkner et al., 2017). The shell acts as a selective barrier that is permeable to bicarbonate and ribulose-1,5-bisphosphate (RuBP), the substrates of Rubisco (Dou et al., 2008). CA dehydrates bicarbonate into CO_2_ in the carboxysome lumen, supplying significant accumulation of CO_2_ in proximity to Rubisco to enhance carbon fixation (Peña et al., 2010). The shell and internal linking proteins are encoded by a *ccmKLMNO* operon, in which *ccmK*, *ccmL*, and *ccmO* encode shell proteins, whereas *ccmM* and *ccmN* encode internal linking proteins for Rubisco packing in the carboxysome lumen (Long et al., 2007).

Deciphering the molecular mechanism underlying carboxysome biogenesis has been the key target for installing functional cyanobacterial CCM in plants, with the aims of supercharging photosynthetic efficiency and improving crop production. Different models have been proposed to illustrate the biogenesis of carboxysomes, one of which, known as the “inside-out” model, suggests that correct packing of Rubisco holoenzymes with the interior component CcmM triggers the formation of a core, followed by the encapsulation of shell proteins to form entire carboxysomes (Cameron et al., 2013; Chen et al., 2013). During this process, Rubisco coalesces into a discrete punctum to form procarboxysome. This assembly pathway indicates the necessity of proper Rubisco assembly and packing in carboxysome biogenesis. However, our understanding of the molecular mechanisms that mediate Rubisco assembly in cyanobacteria and the significance of Rubisco assembly in carboxysome formation is still rudimentary.

In this study, we investigated the in vivo function, spatial localization, and dynamics of RbcX as well as its correlation with carboxysome organization and formation in Syn7942, using molecular genetics, biochemical assays, and live-cell microscopic imaging. We show that depletion of RbcX resulted in not only an increase in Rubisco abundance, but also the perturbance of carboxysome number and size. We also show that RbcX serves as one component of the carboxysome and has a specific association with Rubisco complexes. Our study provides insights into the roles of Syn7942 RbcX in carboxysome assembly.

## RESULTS

### Bioinformatic Analysis Suggests the Functional Divergence of RbcX among Species

**Figure 1.**
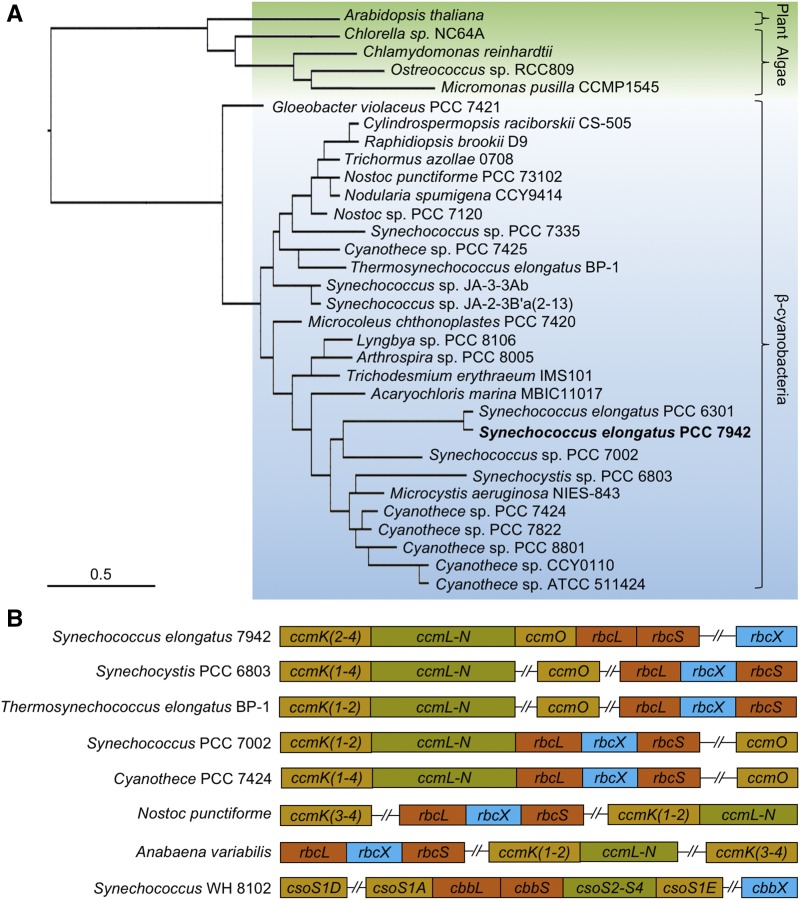
Phylogenetic analysis of RbcX and genomic locations of the *rbcX* genes. A, Molecular phylogeny of RbcX. A total of 32 RbcX sequences from cyanobacteria, algae, and plant were phylogenetically analyzed using PubSEED. Cyanobacterial RbcX sequences are highlighted in blue, and RbcX sequences from plants and algae are highlighted in green. Scale bar, substitutions per site. B, Genomic organization of the *rbcX* genes relative to other carboxysomal genes in cyanobacteria. Compared with other β-cyanobacteria, Syn7942 *rbcX* is not integrated within the Rubisco *rbcLS* operon. *Synechococcus* WH 8102 is classified as an α-cyanobacterium which does not contain *rbcX* but has *cbbX*, a red-type Rubisco activase.

The *rbcX* genes are widespread in cyanobacterial, algal, and plant genomes. Phylogenetic analysis of the chosen RbcX protein sequences from cyanobacteria and their predicted homologs from green algae (such as *Chlamydomonas reinhardtii*) and land plants (such as Arabidopsis [*Arabidopsis thaliana*]) showed that the divergence of RbcX sequences occurs not solely between cyanobacteria, green algae, and plants but also from the primordial cyanobacterium *Gloeobacter violaceus* in the cyanobacterial clade (Fig. 1A). In many cyanobacteria that possess form IB Rubisco, such as *Synechocystis* PCC 6803, *Thermosynechococcus elongatus* BP-1, Syn7002, *Cyanothece* PCC 7424, *Nostoc punctiforme*, and *Anabaena variabilis*, the *rbcX* gene is located between the *rbcL* and *rbcS* genes to form the *rbcLXS* operon (Fig. 1B). This is a common feature in cyanobacterial genomes that clustering of genes encodes structurally related components. Two exceptions are Syn7942 and Syn6301, in which *rbcX* is not positioned in the *rbcLS* operon (Fig. 1B). RbcX from Syn7942 and Syn6301 possess a high sequence similarity (94%; Fig. 1A; Supplemental Fig. S1). This is in striking contrast to the low sequence similarity of RbcX proteins among different species (57.2% overall, 12.5% identity and 44.7% pseudoidentity; Supplemental Fig. S1). In particular, the Syn7942 RbcX shares 46% overall sequence similarities with RbcX from Syn7002 and 50% with that from the thermophilic cyanobacterium *T. elongatus*, in which RbcX proteins have been demonstrated to be key for Rubisco assembly (Onizuka et al., 2004; Tarnawski et al., 2008). Two conserved regions were found at the N terminus of RbcX (8–35 aa and 68–110 aa; Supplemental Fig. S1), which were revealed to be responsible for Rubisco assembly (Saschenbrecker et al., 2007). By contrast, the C-terminal regions lack sequence similarities, consistent with the previous study (Tarnawski et al., 2008). Collectively, the locus divergence and low sequence similarity signify the species-specific roles of RbcX in Syn7942 (and Syn6301).

### Generation and Characterization of the Syn7942 Mutant with Inactive *rbcX*

**Figure 2.**
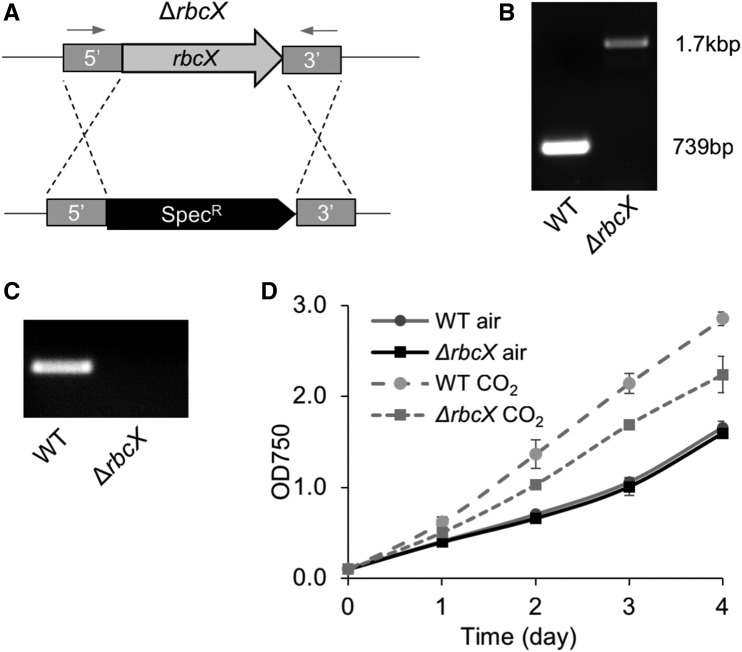
Construction and characterization of the Syn7942 ∆*rbcX* mutant. A, A graphical depiction of the genetic deletion of *rbcX* by replacing the complete open reading frame of *rbcX* with the spectinomycin-resistant gene. Arrows indicate the positions of primers for genotyping gene insertions. B, PCR verification of the full segregation of ∆*rbcX* mutants. The *rbcX* gene is 739 bp, and the spectinomycin-resistant gene is 1.7 kb. C, RT-PCR of the *rbcX* transcript in Syn7942 wild type (WT) and ∆*rbcX* mutant. D, Growth of wild type and ∆*rbcX* mutant in air and 5% CO_2_. Data are represented as mean ± sd. Three independent cell cultures were analyzed.

Previous studies have investigated the RbcX function in different cyanobacterial strains by insertional inactivation of the *rbcX* gene. RbcX in Syn7002 was found to be essential for cell survival (Onizuka et al., 2004), whereas no detectable phenotypic differences were found in the reported Syn7942 *rbcX* knockdown mutant, in which the *rbcX* gene sequence was only partially deleted and inactivated (Emlyn-Jones et al., 2006b). We utilized a different genetic strategy to ensure the complete deletion of the *rbcX* gene in Syn7942. The *rbcX* gene in the wild-type Syn7942 genome was replaced by the spectinomycin resistance gene through homologous recombination via ∼800 bp sequences upstream and downstream of *rbcX* (Fig. 2A). Fully segregated *rbcX* knockout (∆*rbcX*) transformants were readily obtained, as confirmed by PCR and sequencing (Fig. 2B). No *rbcX* mRNA was detected in the ∆*rbcX* mutants by reverse transcription PCR (RT-PCR) analysis (Fig. 2C), confirming the complete deletion of the *rbcX* gene.

The ∆*rbcX* homozygous mutant survives in both air and high-CO_2_ (5%) conditions. The growth rate of the ∆*rbcX* mutant is equivalent to wild type under ambient air conditions (Fig. 2D), consistent with previous observations (Emlyn-Jones et al., 2006b), indicating that RbcX is not essential for cell growth in Syn7942. In air supplemented with 5% CO_2_, both wild type and ∆*rbcX* mutant exhibit increased cell growth rate compared with that of the cells growing in air, whereas an increase in the cell growth of the ∆*rbcX* mutant appears less significant than that of wild type (Fig. 2D). The different responses to changes in the level of CO_2_ between the ∆*rbcX* mutant and wild type suggest the involvement of RbcX in carbon fixation.

### The Effects of *rbcX* Deletion on Rubisco Content and Activity

**Figure 3.**
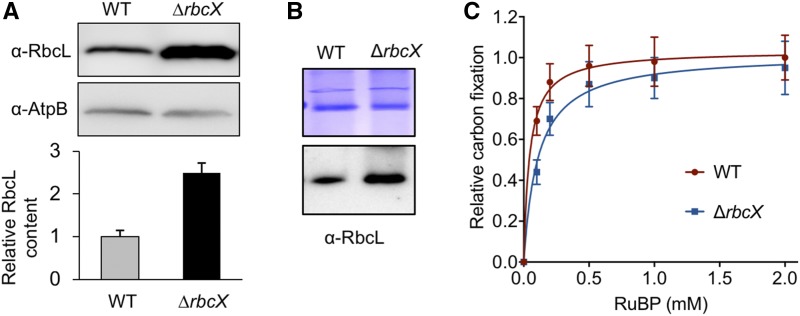
Rubisco content and activity in ∆*rbcX* and wild type Syn7942 strains. A, Immunoblot analysis of total protein extracts using anti-RbcL antibody shows ∼2.5-fold increase in Rubisco abundance in ∆*rbcX* cells compared to that in wild type (WT) cells. Data are represented as mean ± sd. Beta subunit of ATP synthase (AtpB) was used as a loading control. Gels are representative of three independent experiments. B, Rubisco holoenzymes were shown in the soluble fraction of protein extracts from the wild type and the ∆*rbcX* mutant by blue native-PAGE (top) and immunoblot analysis using anti-RbcL antibody (bottom). C, Kinetics of whole-cell carbon fixation activity of the ∆*rbcX* mutant relative to that of wild type (*V*_max_). Data are represented as mean ± sd. Three independent cell cultures were analyzed.

We measured the cellular Rubisco content and activity in the *∆rbcX* mutant growing in air. Quantification analysis based on sodium dodecyl sulfate-polyacrylamide gel electrophoresis (SDS-PAGE) and immunodetection using anti-RbcL antibody, normalized by the beta subunit of ATP synthase content, showed that the total RbcL amount in protein extracts was increased more than two folds in the ∆*rbcX* mutant compared with that in the wild type (Fig. 3A), indicating the enhancement of Rubisco content. In addition, the solubility of RbcL was tested by fractionation of the total protein extracts using centrifugation at 12,000 rpm for 10 mins. Equal volumes of the supernatant and pellet resuspension that was resuspended in the same volume as the supernatant was loaded on SDS-PAGE, followed by immunoblot analysis using anti-RbcL antibody. RbcL was only detected in the supernatant and not in the pellet in both wild type and *∆rbcX* strains (Supplemental Fig. S2), suggesting that the overwhelming majority of Rubisco exists in the soluble fraction. The Rubisco content in the soluble fraction was then measured by using 3%­–12% Bis-Tris Native-PAGE and immunodetection using anti-RbcL antibody. A band with *M*_r_ ∼ 550 kD representing the Rubisco holoenzyme RbcL_8_S_8_ was identified in both the wild type and ∆*rbcX* mutant (Fig. 3B), indicating that Rubisco peptides could be properly folded and assembled without RbcX. This finding is in agreement with the previous observation that RbcX is not required for assembly of Syn7942 Rubisco in tobacco chloroplasts (Occhialini et al., 2016).

We further examined the carboxylation activity of cells using maximum carbon fixation rate (*V*_max_) as reported previously (Sun et al., 2016). The Rubisco activity kinetics shows a similar *V*_max_ for the wild type and ∆*rbcX* mutant (Fig. 3C), suggesting that the activity of the Rubisco complex was not impeded by loss of RbcX.

### Carboxysome Formation Was Interfered in the ∆*rbcX* Mutant

It was shown that Rubisco is densely packed into an ordered matrix inside β-carboxysomes (Faulkner et al., 2017). This crystalline packing of Rubisco is important for the initiation of carboxysome shell encapsulation (Cameron et al., 2013; Chen et al., 2013). Given that RbcX is not pivotal for Rubisco assembly in Syn7942, what are the exact functions of RbcX in Syn7942? Is it involved in carboxysome biogenesis? To address these questions, we used the RbcL-eGFP strain to determine the subcellular positioning and biosynthesis of carboxysomes in vivo (Savage et al., 2010; Cameron et al., 2013; Sun et al., 2016). The ∆*rbcX* construct was then introduced into the RbcL-eGFP strain (Sun et al., 2016). The fully segregated ∆*rbcX* transformants in the RbcL-eGFP background was obtained (∆*rbcX/*RbcL-eGFP), as verified by PCR.

**Figure 4.**
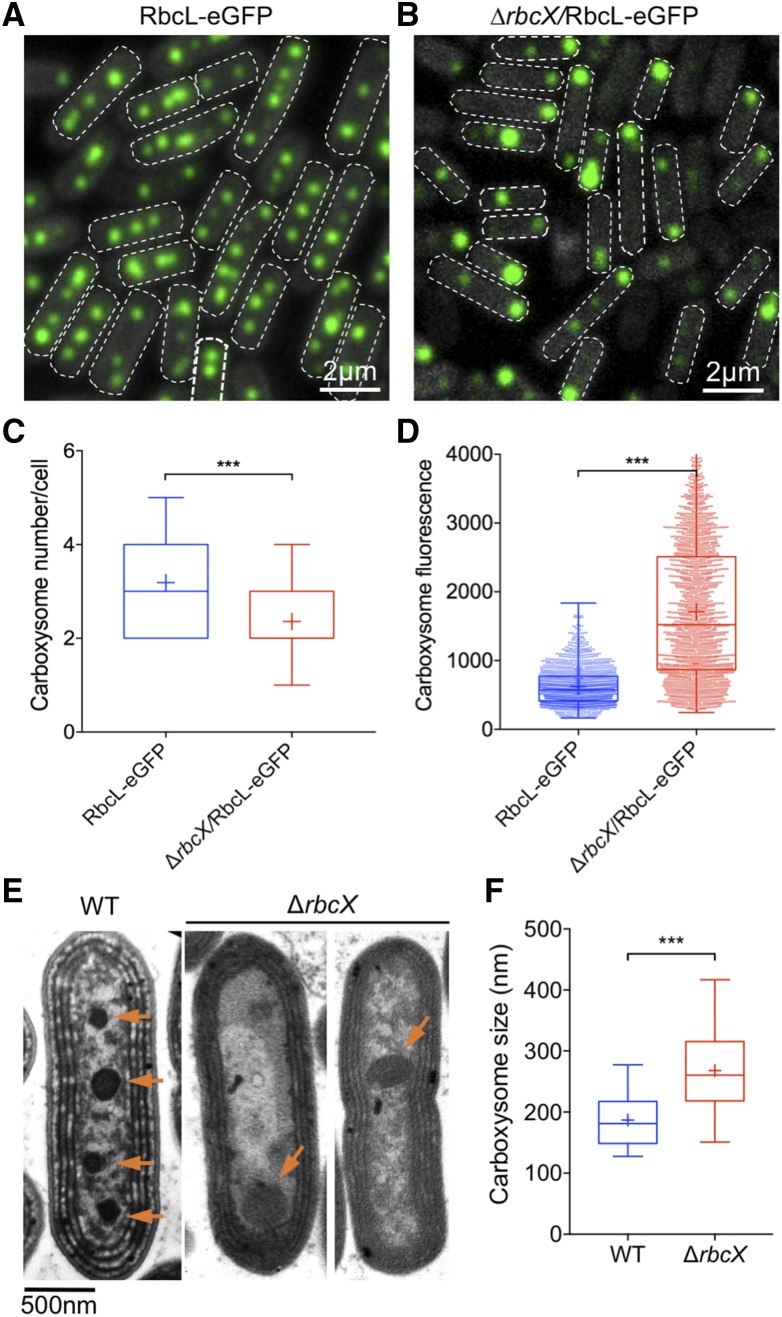
Organization and distribution of carboxysomes in Syn7942 RbcL-eGFP and ∆*rbcX*/RbcL-eGFP cells. A and B, Confocal microscopy images of the RbcL-eGFP and ∆*rbcX*/RbcL-eGFP cells. Green signal represents the RbcL protein encapsulated in carboxysomes, and the dashed white line represents the boundary of the cell. C, Quantification analysis of confocal images shows the average numbers of carboxysomes per cell in RbcL-eGFP, and ∆*rbcX*/RbcL-eGFP strains are 3.2 ± 1.4 and 2.3 ± 1.0, respectively (mean ± sem, *n* = 360, two-tailed Student’s *t* test, *P* < 0.0001). D, Quantification analysis of individual carboxysome shows that deletion of the *rbcX* gene results in a 2.7-fold increase in the average fluorescence intensity per carboxysome and more remarkable heterogeneity of carboxysome size (mean ± sem, *n* = 1,800, two-tailed Student’s *t* test, *P* < 0.0001. The RbcL content per cell, based on carboxysome number per cell and the fluorescence intensity of individual carboxysomes, was found to have a 2-fold increase in the ∆*rbcX* mutant. E, Thin-section transmission electron microscopy images of Syn7942 wild-type and ∆*rbcX* cells. The number of carboxysomes in ∆*rbcX* cells is reduced. Orange arrows point to the carboxysomes. F, Statistical characterization of the electron microscopy images reveals that the median carboxysome diameter increases from 156.9 ± 42.4 nm to 282.6 ± 85.7 nm (mean ± sem, *n* = 30, two-tailed Student’s *t* test, *P* < 0.0001). *** Indicates *P* < 0.0001.

Then GFP signal of the *∆rbcX/*RbcL-eGFP cells was visualized by live-cell confocal fluorescence microscopy to characterize carboxysome biogenesis and organization in vivo, using the RbcL-eGFP strain as the control. Figures 4A and 4B display the organization of carboxysomes containing RbcL-eGFP in the wild type and ∆*rbcX* mutant, respectively. In wild type, three to four carboxysomes are evenly distributed along the longitudinal axis of the cell (Fig. 4A), similar to previous reports (Savage et al., 2010; Sun et al., 2016). By contrast, the carboxysome number was reduced in the *∆rbcX/*RbcL-eGFP cells. Image analysis confirmed that the average number of carboxysomes per cell was reduced from 3.2 to 2.3 (Fig. 4C). In addition, the fluorescence intensities of individual carboxysomes in the *∆rbcX/*RbcL-eGFP strain present higher heterogeneity, suggesting a remarkable variety of Rubisco content and carboxysome size in the *∆rbcX/*RbcL-eGFP mutant. Often there is a large carboxysome in a polar manner and the remaining small ones are randomly distributed inside the *∆rbcX/*RbcL-eGFP cells, distinct from the even distribution observed in the RbcL-eGFP cells (Supplemental Fig. S3). There is a 2.7-fold increase in the average fluorescence intensity per carboxysome in *∆rbcX/*RbcL-eGFP cells (Fig. 4D; *P* < 0.05, two-tailed Student’s *t* test), suggesting an increase in RbcL content in the ∆*rbcX* mutant background, consistent with immunoblot results (Fig. 3A). All these results revealed that carboxysome number, size, and positioning in Syn7942 are interfered by depletion of RbcX.

The changes in carboxysome number and size in the ∆*rbcX* mutant background were further substantiated by transmission electron microscopy images of Syn7942 wild type and ∆*rbcX* mutant (Fig. 4E). Statistical analysis revealed that the average diameter of wild-type carboxysomes is 156.9 ± 42.4 nm (*n* = 30), consistent with the results obtained from the isolated carboxysomes from Syn7942 (Faulkner et al., 2017), whereas the ∆*rbcX* cells possess larger carboxysomes, 282.6 ± 85.7 nm (*n* = 30) in diameter (Fig. 4F), consistent with the increased average fluorescence intensity per carboxysome in *∆rbcX/*RbcL-eGFP cells (Fig. 4D). These findings support a role of RbcX in carboxysome assembly and organization in Syn7942.

### In Vivo Localization of RbcX and Colocalization with Rubisco

**Figure 5.**
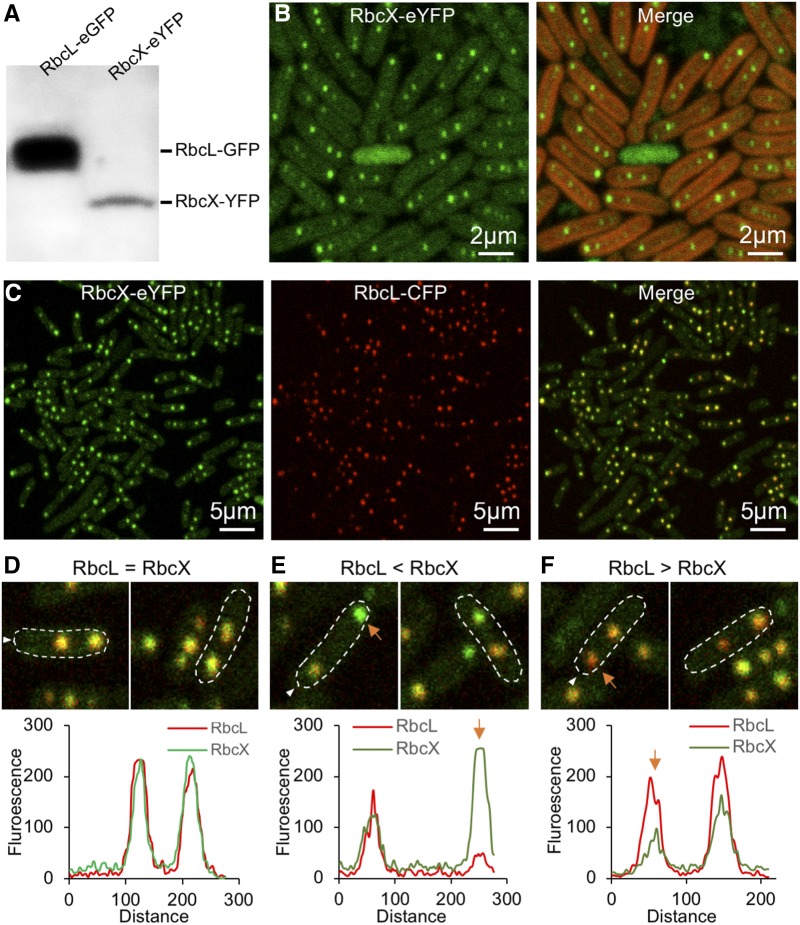
In vivo localization of RbcX and its colocalization with RbcL in Syn7942. A, Immunoblot of soluble protein extracts from RbcL-eGFP (lane 1) and RbcX-eYFP (lane 2) cells using anti-GFP antibody. B, Confocal microscopy images of the RbcX-eYFP cells. Green signal represents the RbcX protein that can be both detected in cytosol and compartmentalized. Red signal represents chlorophyll autofluorescence. C, Confocal microscopy images of the RbcX-eYFP/RbcL-CFP cells (after normalization of RbcX fluorescence). Green channel, eYFP-labeled RbcX; red channel, CFP-labeled RbcL representing Rubisco and carboxysomes; merged channel, colocalization of RbcX and RbcL. D–F, Colocalization analysis reveals three different RbcX-RbcL ratios in the carboxysome. D, 80% of carboxysomes have similar ratios of RbcX and RbcL. E, 10% of carboxysomes present a high content of RbcX (orange arrow). F, 10% of carboxysomes present a low content of RbcX (orange arrow).

RbcX was shown to promote Rubisco assembly by interacting with RbcL in vitro (Saschenbrecker et al., 2007). However, its in vivo localization at the cellular level and the dynamic interaction with RbcL are still not clear. To address these questions, we generated a RbcX-eYFP mutant strain by tagging eYFP at the 3′ end of *rbcX* (Supplemental Fig. S4A). Full genetic segregation of the RbcX-eYFP mutant was confirmed by PCR screening (Supplemental Fig. S4B). The doubling time was 18.14 ± 0.77 h for the RbcX-YFP mutant and 16.82 ± 1.31 h for the wild type (*n* = 4), demonstrating no significant growth defects caused by fluorescence tagging. Immunoblot analysis using anti-GFP antibody identifies a single band with a *M*_r_ of 43 kD, referring to RbcX-eYFP (Fig. 5A). Confirmation of the strain supports that the YFP fluorescence represents the RbcX localization in vivo. Confocal imaging illustrated that RbcX is not only expressed in the cytosol, but also compartmentalized (Fig. 5B), reminiscent of the characteristic carboxysome distribution pattern in vivo (Fig. 4A).

To further clarify if the RbcX fluorescence puncta colocalize with carboxysomes, we generated the RbcL-CFP construct using the same strategy for the RbcL-eGFP construct (Sun et al., 2016; Supplemental Fig. S4C) and transformed it into the RbcX-eYFP strain to produce the RbcX-eYFP/RbcL-CFP double-labeling mutant for colocalization analysis of RbcX-YFP (green) and RbcL-CFP (red; Fig. 5C). Partial segregation of the RbcL-CFP mutant was confirmed by PCR screening (Supplemental Fig. S4D). Confocal fluorescence imaging revealed that RbcX spots appeared predominantly colocalizing with RbcL, suggesting the direct involvement of RbcX in carboxysome assembly and interactions between RbcX and RbcL. Detailed colocalization analysis, based on the merged images, revealed three categories of colocalization patterns of RbcX-RbcL complex in vivo. The principal pattern (80% possibility) was that RbcX and RbcL have similar ratios within a single carboxysome (Fig. 5D), indicative of a stable interaction between RbcX and RbcL. In addition, the RbcX-enriched fluorescent spots (10% possibility; Fig. 5E, orange arrow) and RbcX-less fluorescence spots (10% possibility; Fig. 5F, orange arrow) were also seen. These structures may represent specific assembly intermediates of carboxysomes at different stages during carboxysome biogenesis (Cameron et al., 2013).

**Figure 6.**
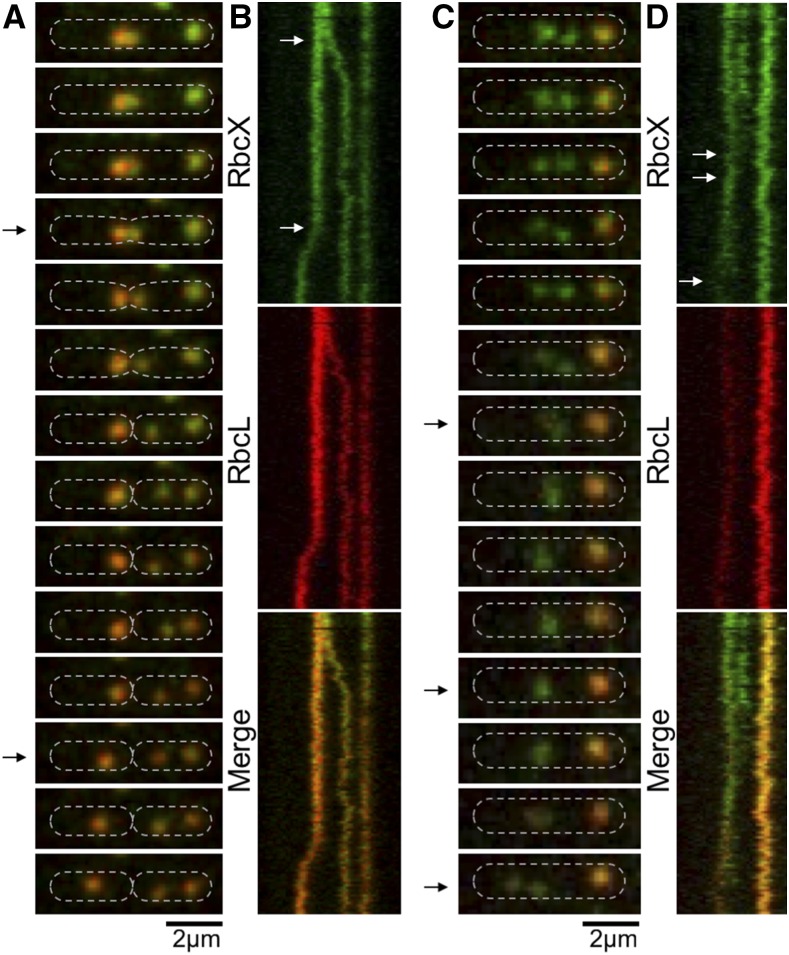
Dynamics of RbcX-Rubisco assembly in live Syn7942 cells using time-lapse confocal fluorescence imaging. A, Time-lapse confocal images of a RbcX-eYFP/RbcL-CFP cell, showing the dynamic locations and interactions of RbcX and Rubisco during the carboxysome birth event and a cell-dividing process. Time interval, 1.25 min. B, Kymographs of RbcX-eYFP (green) and RbcL-CFP (red) assembly. Arrows indicate a carboxysome birth event and a cell-dividing event (bottom), as shown in A. Time interval, 1.25 min. Scale bar, 2 µm. C, Time-lapse confocal images showing the dynamic locations and interactions of RbcX and Rubisco during the fusion and splitting processes of RbcX-containing spots. Time interval, 1.25 min. D, Kymographs of RbcX-eYFP (green) and RbcL-CFP (red) assembly. Arrows indicate a fusion event and a dividing event (bottom) of RbcX-containing spots, as shown in (C). Time interval, 1.25 min. Scale bar, 2 µm.

We further monitored the RbcX-RbcL assembly dynamics using time-lapse confocal fluorescence imaging. Figure 6 shows a carboxysome birth event and the separation of carboxysomes into two daughter cells during cell division. During the course of imaging, RbcX was present in not only the “static” carboxysomes but also the mobile and newly generated carboxysomes (Fig. 6A), suggesting the participation of RbcX throughout the carboxysome biogenesis pathway. This is further confirmed by the kymographs of RbcX-eYFP and RbcL-CFP (Fig. 6B). Figure 6, C and D, illustrates the fusion of two RbcX-enriched spots into one spot. In these spots, the abundance of Rubisco is notably low compared with that in mature carboxysomes. The composition and actual roles of these structures await further investigations. Nevertheless, our results indicated explicitly that RbcX proteins colocalize with carboxysomes in Syn7942 and structurally associate with Rubisco and carboxysomes during cell growth.

## DISCUSSION

Current knowledge about the functions of chaperones in Rubisco assembly was predominantly obtained from in vitro reconstitution experiments or heterologous expression in *E. coli* (Saschenbrecker et al., 2007; Liu et al., 2010; Bracher et al., 2011, 2015; Georgescauld et al., 2014). However, these experimental conditions do not resemble real physiological conditions in cyanobacterial cells, and there are no correlated biological processes taking place, such as the subsequent Rubisco aggregation and carboxysome formation. This study represents our intent of deciphering the physiological action of RbcX in the native host cells, using a combination of bioinformatic, genetic, physiological, biochemical and fluorescence imaging approaches.

We found that inactivation of Syn7942 *rbcX* has no detectable effects on cell growth and Rubisco assembly but could result in an increase in total Rubisco content. Furthermore, we showed that inactivation of *rbcX* could induce defective carboxysome formation, as evidenced by changes in carboxysome size, number, and distribution in vivo, demonstrating that RbcX is functionally involved in carboxysome assembly. It was suggested that the stoichiometry of shell and structural components is a crucial factor in the pathway that leads to the assembly of carboxysomes with the physiological shape and size (Long et al., 2010). It would be interesting to investigate how individual carboxysomal protein is regulated in vivo without RbcX in future studies. Our recent work revealed the well-defined 3D structure of the dense packing of interiors in β-carboxysomes (Faulkner et al., 2017), suggesting the highly regulated β-carboxysome assembly pathway. It is likely that the absence of RbcX interferes with proper Rubisco assembly and packing, leading to defective carboxysome formation and reduced carbon fixation efficiency. Possibly as a compensating strategy adopted by cells, the Rubisco amount is increased, either by sustaining its transcript abundance or its translation or by reducing its proteolytic degradation, to maintain carbon fixation efficiency and cell growth. In agreement with this, our kinetics studies show that *V*_max_ of Syn7942 cell carbon fixation activity is not influenced in the absence of RbcX. This could also explain the similar growth rate between wild type and the ∆*rbcX* mutant. Rubisco content has been shown to be highly regulated by environmental factors, such as light and inorganic carbon (Sun et al., 2016). Similar observations of increased Rubisco content were also reported in the pseudorevertant carboxysome-less mutant deficient in the CcmM protein, the linker protein of Rubisco packing (Emlyn-Jones et al., 2006a). Further investigations would reveal the detailed mechanisms that modulate the levels of Rubisco content.

Our bioinformatic data indicated a divergent function of RbcX in different species (Fig. 1). Based on the exceptional gene locus of *rbcX* in Syn7942 and Syn6301, as well as the high sequence similarities of RbcX and RbcL proteins from Syn7942 and Syn6301 (Fig. 1A; Shih et al., 2016), we expect that RbcX has similar functions in Syn6301 and Syn7942. It was reported that Syn6301 Rubisco can be functionally expressed in *E. coli* only in the presence of the bacterial chaperonins GroEL/GroES (Goloubinoff et al., 1989), whereas many cyanobacterial Rubisco, such as Rubisco from Syn7002, require coexpression of RbcX or Raf1 for proper assembly (Saschenbrecker et al., 2007). These results indicated that RbcX in Syn7942 might not only function at the early stage of Rubisco assembly as proposed for RbcX from Syn7002. Instead, we propose that RbcX may also be involved in the later stage of Rubisco holoenzyme stabilization or adjusting the packing of Rubisco with the assistance of carboxysomal internal linker proteins, and thereby mediating the initiation of carboxysome formation.

Introducing cyanobacterial carboxysomes into plant chloroplasts has been a target for genetic engineering to improve photosynthetic performance (Gonzalez-Esquer et al., 2016; Hanson et al., 2016; Rae et al., 2017). β-carboxysomes from Syn7942 are one of the most well studied in synthetic engineering of plants (Lin et al., 2014a, 2014b; Occhialini et al., 2016). However, reconstituting entire functional and structurally controllable β-carboxysomes in heterologous hosts is still challenging, due in part to the complex mechanisms of Rubisco assembly and carboxysome biogenesis. Although previous work has illustrated that RbcX is not required for assembly of Syn7942 Rubisco installed into tobacco chloroplasts (Occhialini et al., 2016), whether RbcX is needed for Rubisco packing and carboxysome formation in engineered chloroplasts awaits further investigation. Nevertheless, our recent work showed that dense packing of Rubisco proteins was achievable in the reconstituted functional β-carboxysome-like structures produced from *E. coli* in the presence of RbcX, suggesting the biological importance of RbcX (Fang et al., 2018). This study provides an advanced understanding of the function of RbcX from Syn7942 in carboxysome formation, which will inform the design and engineering of functional carboxysome structures in plants for enhanced carbon fixation and agricultural productivity.

### CONCLUSION

In this study, we applied molecular genetics, physiological assays, and live-cell fluorescence microscopy to investigate the in vivo roles of Syn7942 RbcX. Unlike many cyanobacterial species, the *rbcX* gene in Syn7942 is distant from the Rubisco gene operon, implying the species-dependent functions of RbcX. Depletion of RbcX has effects on Rubisco content, carboxysome formation, and in vivo localization but does not affect Rubisco holoenzyme formation. Exploration of RbcX localization and dynamics in Syn7942 revealed that RbcX may act as a component of the Rubisco complex and carboxysome, shaping Rubisco complexes, organizing Rubisco packing and mediating carboxysome assembly. Our study provides insights into the physiological function of RbcX in the cell and offers a pipeline for evaluating the effects of auxiliary proteins on Rubisco biogenesis and carboxysome assembly. A comprehensive understanding of the mechanism governing Rubisco and carboxysome biogenesis is of significant importance for re-engineering Rubisco and carboxysomes to improve plant productivity.

## MATERIALS AND METHODS

### Bacterial Strains, Growth Conditions, and Physiology

The cyanobacterium *Synechococcus elongatus* PCC 7942 (Syn7942) was maintained on solid BG11 medium (Rippka, 1988) at 30°C with constant 30 μmol quanta m^−2^ s^−1^ illumination provided by LED lamps. Liquid cultures were grown at 30°C under constant 40 μmol quanta m^−2^ s^−1^ illumination in BG11 medium in culture flasks with constant shaking either in air or in a cabinet supplemented with high CO_2_ (5%). Where appropriate, kanamycin, apramycin, or spectinomycin was added to the medium at a final concentration of 30 μgmL^−1^, 50 μgmL^−1^ or 25 μgmL^−1^ individually.

### Sequence Alignment

SEED (pubseed.theseed.org) database and the comparative genomics platform was used for retrieval and alignment of the corresponding genes and genomic regions from cyanobacteria, green algae (*Chlamydomonas reinhardtii*) and Arabidopsis (*Arabidopsis thaliana*). The software has been made available as open source software released under the GNU public license from the FTP site (ftp://ftp.theseed.org/SEED). The selected genes were aligned and used for phylogenetic tree reconstruction by application of SEED-integrated software (ClustalW 1.83; Overbeek et al., 2005, 2014).

### Generation of Constructs and Syn7942 Transformation

To inactivate *rbcX* in Syn7942, a 2,046-bp fragment containing the *rbcX* (*synpcc7942_1535*) open reading frame and 700- to 800-bp homologous sequences upstream and downstream of *rbcX* was amplified from Syn7942 genomic DNA using the primers rbcXF (5′-AAGCAGGTTGGCAGCCTATC-3′) and rbcXR (5′-TCGCTGTCATCAAGGCATCG-3′) and cloned into the pGEM-T Easy vector (Promega) yielding pGEMrbcX. A fragment containing the *aadA* gene encoding spectinomycin resistance and flanking linker regions was amplified from the pIJ778 plasmid using the primers rbcXkoF (5′-CACGGCTGGATGCAATTTATGGGTACAGCCTCTAGGATGATTCCGGGGATCCGTCGACC-3′) and rbcXkoR (5′-GCGGCAGGCCCTTCAAAATCAACGTGTTGAACAATTTCATGTAGGCTGGAGCTGCTTC-3′). The fragment was then used to replace *rbcX* in pGEMrbcX by electroporation, using the Redirect strategy (Gust et al., 2004), to generate the knockout construct pGEM∆*rbcX*. The RbcX-eYFP fusion construct was created by inserting the *eyfp:apramycin* fragment onto the 3′ end of *rbcX* following the method described previously (Liu et al., 2012; Sun et al., 2016; Casella et al., 2017). The RbcL-CFP construct was created by inserting the *cfp:kanamycin* fragment onto the 3′ end of the *rbcL* gene. The final plasmids were transformed into Syn7942 wild-type or mutant cultures according to the description in the results, following the method described earlier (Golden, 1988). Segregation analysis was done by PCR genotyping using the primers rbcXkosegF (5′-GATAAGTTAATTGCGGTCTA-3′) and rbcXkosegR (5′-TTCCGTCAGCAGCCAAGGAT-3′) for ∆*rbcX*::Spec, the primers rbcXYFPsegF (5′-ATGCTTCTAATGCCTCCCA-3′) and rbcXYFPsegR (5′-CGTCAGCAGCCAAGGATAG-3′) for RbcX-eYFP::Apra, and the primers rbcLGFPsegF (5′-CGTGAAGCTGGCAAGTGG-3′) and rbcLGFPsegR (5′-GGAGGCAGGTACGAGAAAGT-3′) for RbcL-CFP::Kan.

### RNA Isolation, cDNA Synthesis, and Semiquantitative RT-PCR

Cells were collected by centrifugation (6,000*g*, 5 min) in 50-mL centrifuge tubes and concentrated in 1 mL growth medium and transferred to a 1.5-mL microcentrifuge tube. The cells were recentrifuged (10,000*g*, 1 min), and the pellet was used for total RNA isolation using TRIzol reagent protocol (Invitrogen). The RNA was digested with 4 units of DNase (RQ1 RNase-free DNase, Promega) according to the manufacturer’s instructions before cDNA synthesis to avoid amplifying genomic sequences. The digest was extracted with an equal volume of phenol:chloroform (5:1 [w/v]), and the RNA was precipitated by centrifugation after a 40-min incubation at −20°C in the presence of 75 mm sodium acetate buffer (pH 5.2) and 75% (v/v) ethanol.

First-strand cDNA was synthesized using Tetro cDNA synthesis kit (Bioline), conducted as described in McGinn et al. (2003). Primers used to analyze the *rbcX* transcript were the same as described previously (Emlyn-Jones et al., 2006b).

### Transmission Electron Microscopy

The cultures of ∆*rbcX*::Spec^R^ mutant and wild-type Syn7942 cells were pelleted and fixed for 1 h with 4% paraformaldehyde and 2.5% glutaraldehyde (v/v; Agar scientific) in 0.05 m sodium cacodylate buffer at pH 7.2. Cells were then postfixed with 1% osmium tetroxide (w/v; Agar scientific) for 1.5 h, dehydrated with a series of increasing alcohol concentrations (30% to 100%), and embedded in resin. Thin sections of 70 nm were cut with a diamond knife and poststained with 4% uranyl acetate (w/v) and 3% Reynolds’ lead citrate (w/v). Images were recorded using an FEI Tecnai G2 Spirit BioTWIN transmission electron microscope.

### Confocal Microscopy and Image and Data Analysis

Preparation of Syn7942 cells for confocal microscopy was performed as described earlier (Liu et al., 2012; Casella et al., 2017). Confocal laser scanning microscopy used a Zeiss LSM710 or LSM780 with a 63× or 100× oil-immersion objective. GFP, YFP, and CFP were excited at 488 nm, 512 nm, and 440 nm, respectively. Live-cell images were recorded from at least five different cultures. All images were captured with all pixels below saturation. Image analysis was carried out using Fiji software and Image SXM. Graphs were created using GraphPad Prism 7. Box plots contain error bars that display mean ± sem. The line in the box is plotted at the median and “+” represents the mean. Other results are presented as mean ± sd. Significance was assessed using a two-tailed Student’s *t* test.

### In Vivo Carbon Fixation Assay

The whole-cell carbon fixation assay was performed as descried previously (Sun et al., 2016). Cells were harvested at the exponential phase and then resuspended in Rubisco assay buffer (100 mm EPPS, pH 8.0, and 20 mm MgCl_2_). Cell density was calibrated to OD_750_ = 4. Cell cultures prepared in assay buffer with the same cell density were incubated with NaH^14^CO_3_ (final concentration at 25 mM) at 30°C for 2 min and then permeabilized by mixing with alkyltrimethylammonium bromide (final concentration at 0.03% [w/v]; Sigma-Aldrich). RuBP (Sigma-Aldrich, purity ≥99.0%) was then added in the samples with a range of concentrations (0–2.0 mM) to initialize the fixation. This enabled us to determine the maximum fixation velocity (*V*_max_). The duration of fixation was set to 5 min to balance between sufficient/accurate counting and minimum exposure of carboxysomes after cell permeabilization at 30°C. The reaction was terminated by adding 10% (v/v) formic acid, as reported previously (Badger and Price, 1989; Hudson et al., 1992). Samples were then dried on heat blocks at 95°C to remove unfixed NaH^14^CO_3_, and the pellets were resuspended in distilled water in the presence of scintillation cocktail (Ultima Gold XR; Perkin-Elmer). Radioactivity measurements were carried out using a scintillation counter (Tri-Carb; Perkin-Elmer). Raw readings were processed to determine the amount of fixed ^14^C, calibrated by blank cell samples without providing RuBP, and then converted to the total carbon fixation rates. *V*_max_ was calculated by Michaelis-Menten plot using GraphPad Prism. For each experiment, at least three independent cell cultures were prepared. Significance was assessed using a two-tailed Student’s *t* test.

### Protein Extraction from Syn7942

Protein extracts were prepared from 50 mL cyanobacterial cultures growing to cell densities (measured by *A*_750_) around 1. The cells were harvested by centrifugation (6,000*g*, 10 min), washed in Tris-EDTA buffer (20 mm Tris-HCl, 0.5 mm EDTA, pH 8.0), centrifuged, and resuspended in Tris-EDTA buffer containing protease inhibitor cocktail (Promega). The cells then were broken by sonication at 4°C followed by 1% Triton X-100 (v/v) treatment and centrifugation (4,000*g*, 10 min at 4°C) to remove unbroken cells. The total cellular extracts were separated into soluble and insoluble fractions by centrifugation at 12,000 rpm for 10 min, and the pellet was resuspended into buffer with the same volume of supernatant.

### SDS-PAGE, Blue Native-PAGE, and Immunoblot Analysis

According to the demand of the experiment, the corresponding fraction was loaded on 10% SDS-PAGE or 3%–12% Bis-Tris Native-PAGE (Invitrogen). Gels were blotted onto a polyvinylidene difluoride membrane (Bio-Rad). The membrane was immunoprobed using rabbit polyclonal antisera against RbcL and beta subunit of ATP synthase (Agrisera) and then goat anti-rabbit horseradish peroxidase-conjugated secondary antibody (Agrisera) or by using GFP tag monoclonal antibody (Invitrogen) and then rabbit anti-mouse horseradish peroxidase-conjugated secondary antibody (Agrisera). Immunoreactive polypeptides were visualized by using the western ECL blotting substrate (Bio-Rad). Signal quantification was carried out using Fiji. For each experiment, at least three independent cell cultures were performed.

### Accession Numbers

Sequence data for this article can be found in the KEGG or CyanoBase databases under the following accession numbers*:* Synpcc7942_1535 (*rbcX*) and Synpcc7942_1426 (*rbcL*).

### Supplemental Data

The following supplemental materials are available.

**Supplemental Figure S1.** Sequence alignment of RbcX proteins
**Supplemental Figure S2.** Solubility of Rubisco by fractionation of protein extracts
**Supplemental Figure S3.** Spatial organization of carboxysomes in Syn7942 RbcL-eGFP and ∆*rbcX*/RbcL-eGFP cells
**Supplemental Figure S4.** Construction and characterization of RbcX-eYFP and RbcL-CFP mutants
